# The Risk Factors and Preventive Strategies of Poor Knee Functions and Osteoarthritis after Anterior Cruciate Ligament Reconstruction: A Narrative Review

**DOI:** 10.1298/ptr.R0028

**Published:** 2023-11-25

**Authors:** I Putu Gde Surya ADHITYA, Ida KURNIAWATI, Ryuichi SAWA, Tabita Febyola WIJAYA, Ni Putu Aprilia Chintya DEWI

**Affiliations:** ^1^Department of Physical Therapy, College of Medicine, Universitas Udayana, Indonesia; ^2^Department of Histology, Faculty of Medicine and Health Sciences, Universitas Warmadewa, Indonesia; ^3^Department of Physical Therapy, Faculty of Health Science, Juntendo University, Japan; ^4^Bachelor and Professional Program of Physical Therapy, College of Medicine, Universitas Udayana, Indonesia

**Keywords:** Anterior cruciate ligament reconstruction, Injury prevention, Poor knee function, Post-traumatic osteoarthritis, Rehabilitation

## Abstract

Anterior cruciate ligament reconstruction (ACLR) is the standard surgical treatment for ACL injury, which typically uses a graft to replace the torn ligament in the knee that uses small incisions with minimally invasive surgery. The optimal knee functions following ACLR depend on rehabilitation processes before and after the surgery. Knee function is the ability of the knee to perform various types of functional movements like walking, squatting, running, jumping, and pivoting where patients expect to achieve maximum knee function or at least more than 80% of its initial condition before the injury to avoid being categorized as poor knee function after ACLR. Patients use patient-reported outcome measures to collect data on their health status and quality of life after ACLR. Post-traumatic osteoarthritis (PTOA) is a type of OA that manifests in local cartilage injury caused by chondrocyte death, and matrix dispersion occurs following a joint injury like ACL injury. Gender, time from injury to surgery, and graft type were considered as risk factors for poor knee function after ACLR, while overweight, meniscus tear, and cartilage defect as risk factors for PTOA. However, age is an internal risk factor for both poor knee function and PTOA following ACLR. This review suggests several strategies to prevent both conditions, including a pre-operative program, comprehensive rehabilitation, body weight control, and return to sport (RTS) consideration based on physical capacity, proper time, and psychological readiness.

**T**he anterior cruciate ligament (ACL) injury is a serious knee injury among athletes in pivoting sports including soccer, basketball, handball, rugby, and futsal^1)^. In the United States, an estimated 200,000 ACL injuries occur yearly; up to 90% of patients choose to get an ACL reconstruction (ACLR)^2)^. Another prospective study showed that about 100,000 ACL reconstructions are carried out annually in the United States, making them one of the most frequent sports medical treatments^3)^. Orthopedic surgeons usually recommend ACLR to patients who cannot return through non-operative management, those who have physically demanding occupations, and those who are active and want to resume participation in high-impact physical activities such as pivoting, cutting, or jumping sports^4)^. Additional indications for ACLR are associated with other ligament injuries, meniscus tears, and articular cartilage defects^5)^.

ACLR is the standard surgical treatment for ACL injury, which typically uses a graft (piece of tissue, such as hamstring or patella tendons) to replace the torn ligament in the knee that uses small incisions with minimally invasive surgery^6)^. However, the optimal knee functions following ACLR depend on rehabilitation processes before (*prehabilitation*) and after the surgery. The knee function is defined as the ability of the knee joint to flex and extend for pretty much any form of daily and sports movements (such as walking, running, stairs climbing, and squatting) without any problems^5)^. Most of the literature suggests that 1–2 months of prehabilitation resulted in a faster recovery for quadriceps muscle strength and that 6–12 months of rehabilitation required for the return to sport (RTS)^7)^. Insufficient rehabilitation may cause several problems, such as poor knee function and osteoarthritis following ACLR.

Poor knee function may increase the possibility of later knee problems and osteoarthritis through high-impact physical activities with unusual knee joint loading patterns. Following ACLR, more than 50% of athletes cannot return to their preinjury level because of poor knee function^8)^. Higher age and female gender are reported to increase the risk for poor knee functions, while early surgery after ACL injury could reduce the risk for poor knee functions^9)^. The meniscus concomitant injury requires the meniscectomy during ACLR that cannot be ignored as the cause of knee osteoarthritis^10)^. In addition, poor knee functions may increase the risk for repetitive injuries during sports activities, which damage the meniscus and cartilage, leading to early degeneration of knee cartilage^11)^.

Therefore, the purpose of this article is to describe general pictures of surgical procedures and the risk factors and preventive strategies for poor knee functions and osteoarthritis after ACLR. This article will outline the definition of ACLR, what measurements are used to measure knee functions and osteoarthritis, and the evaluation of the risk factors and rehabilitation strategies to prevent poor knee functions and osteoarthritis following ACLR.

## Anterior Cruciate Ligament Reconstruction (ACLR)

ACLR is the current standard-of-care surgical treatment for ACL tears in sports medicine. ACLR is the surgery to replace the torn ACL with the grafted tendon in the knee that uses small incisions^12)^. Orthopaedic surgeons now perform ACL reconstructions rather than the repairs due to the ACL’s poor ability to heal, which has been suggested clinically and supported by several in vitro and in vivo studies^13)^. A successful ACLR absolutely needs the right technique and important biomechanical and biological procedures^14)^. This section noted a few important concerns: the graft types of ACLR and the rehabilitation approaches following ACLR.

## Graft Types of ACLR

ACL reconstruction goals are to replicate the insertional construction of the biological ACL, to achieve an appropriate level of strength, to provide secure initial fixation, and to encourage rapid revascularization and maturation^14)^. A vital component of graft selection is to minimize donor site morbidity. Due to the relatively low rate of incident morbidity at the donor site compared to autograft, the allograft is a popular alternative. Each of these graft types has limitations and advantages, and they may be examined based on biomechanical characteristics, donor site morbidity, fixation characteristics, complications, and outcomes^15)^. There are two major graft choices for ACLR, namely, autografts and allografts. An autograft is a tissue obtained from another patient’s body, while an allograft is obtained from a tissue donor. Hamstring (HS), bone–patellar tendon–bone (BPTB), and quadriceps tendon (QT) are commonly used as the autograft^16)^. In situations of revision reconstruction, the choice of the graft should be tailored to the patient’s anatomy, graft utilized in prior ACLR, age, sport, and level of competitiveness. Autografts continue to be the graft of preference in patient populations due to the higher failure rates, higher costs, and risk of repeated rupture associated with ACL allograft reconstructive surgery^17)^. Yet, in most situations, it can be said that autograft is preferable to allograft ACL reconstruction since it uses non-irradiated grafts and is as safe but more expensive^18)^.

### Bone patellar tendon bone (BPTB) autograft

BPTB grafts are the most commonly used autografts for ACL reconstruction, showing excellent results in functional activities^15)^. The surgeon usually took 10 mm wide and 4 mm thick BPTB as the standard graft, for which the maximum workload to failure has been determined to be 2,977 N, greater than native ACL^16)^. Due to its thickness and the bone plugs linked to both ends, the BPTB graft is not suitable for double-bundle ACLR^15)^.

The native ACL and BPTB grafts are similar in length, which is the most similar graft to the native ACL compared to the other grafts. Additionally, where the ACL is linked to the bone, the bone segments on the graft’s end can be positioned directly on the bone itself, resulting in a bone-to-bone healing process and one of the most powerful healing techniques known^19)^. There are several drawbacks to choosing this graft to take into account. Because a portion of the patella’s bone and nearly one-third of its tendon were removed during the graft procedure, the bone and tendon are slightly weakened. Moreover, this may cause anterior knee pain while doing kneeling exercises and increase the likelihood of patellar fractures or patellar tendon tears after ACLR^20)^.

### Hamstring tendon (semitendoneus and gracilis tendon) autograft

Hamstring autografts (semitendinosus and gracilis muscle tendons) can be the alternative option for ACLR. Hamstring tendon autografts are frequently folded on themselves, usually quadrupled, to increase graft diameter and strength to mimic the native ACL. The advantages of using the hamstring tendon compared to patellar tendon autografts are hamstring autograft is collected by a tiny skin incision, resulting in low donor site morbidity and reduced extensor mechanism dysfunction^21)^. However, there are several drawbacks, including unpredictable graft sizes, higher failure rates in certain patient populations, inadequate knee flexion, and prolonged healing times^16)^.

### Quadriceps tendon autograft

The quadriceps tendon autograft is a possible alternative for ACLR based on the literature that found its benefits of adaptability in both the first surgery and revision setting^22)^. The quadriceps tendon can be extracted with or without a bone block as a partial-thickness or full-thickness reconstruction. With comparable patient-reported outcomes, BPTB harvest is more damaging to the tendon than quadriceps tendon harvest. Lower rates of anterior knee pain compared to BPTB, comparable graft size to BPTB, knee stability, superior flexor strength to the hamstring tendon, and high functional scores are all benefits of quadriceps tendon graft^23)^. Nonetheless, there are some drawbacks to quadriceps tendon autografts, such as insufficient graft strength, the possibility of suprapatellar pouch injury, and the potential to further deteriorate quadriceps strength in patients who already have weak quadriceps^24)^.

### Allograft

Common allografts utilized in ACLR include the Achilles tendon, tibialis anterior and posterior, patellar ligament, hamstrings, fascia latae, and peroneus longus^25)^. Minimal donor-site morbidity is essential in the use of allografts. Allografts also offer larger graft sizes, a low incidence of arthrofibrosis, a sped-up healing process, and an improved overall health-related quality of life. In some cases, especially in younger patients, allografts can be helpful, such as knee injuries involving several ligaments, ACLRs with few options for autografts after multiple revisions, patient choice, and circumstances in which the autograft tissue is insufficient^26)^. However, choosing the type of graft may be challenging because allografts for ACLR have unique problems. The main drawbacks include increased failure rates in young and active athletes, higher costs, delayed graft integration, and potential disease transmission (bacterial, viral, and prion)^27)^.

## Rehabilitation Approaches Following ACLR

The rehabilitation plan has a significant role in how quickly and safely a person can reach their pre-injury level of function or RTS activities after ACL reconstruction.

### Week 1 (phase 1)

The important objectives in the first week of ACLR are managing pain, edema, and inflammation, restoring neuromuscular function to prevent postoperative problems, such as muscle atrophy and arthrofibrosis, and restoring range of motion^28)^. Cryotherapy is clinically helpful, in addition to exercise, elevation, postsurgical compression wraps, and pain medication, to significantly decrease postsurgical pain^29)^. Following ACL reconstruction, immediate ROM recovery (with a focus on full extension) improves pain, cartilage homeostasis, patellofemoral issues, quadriceps atrophy, arthrofibrosis, and altered gait^30)^. The patella should be mobilized in many directions because patellar immobility reduces the range of motion and inhibits the quadriceps. A changed gait pattern following ACLR is related to pain, edema, lack of range of motion, and quadriceps weakness in the early rehabilitation phase^31)^.

### Week 2 to 9 (phase 2)

Continuing cryotherapy can help improve postoperative problems, such as inflammation, swelling, and chronic pain, which are usually the main causes of ROM restriction and muscle atrophy^32)^. The ROM of knee extension and patellar mobilization should be maintained, and the ROM of knee flexion can be increased gradually to avoid arthrofibrosis after ACLR^28)^. Isotonic strength training with a focus on endurance in caution ranges (i.e., open-chain: 90–40 degrees and close-chain: 0–60 degrees) considerably improves quadriceps strength while having no negative impacts on the graft and tendons that cause knee laxity and anterior knee pain^33)^. Phase 2 still requires gait training, even if a defensive gait pattern appears normal at first sight, on a flat surface without crutches or a treadmill. Phase 2 rehabilitation of ACLR should specifically include treadmill walking, ergometer cycling, swimming starting in week 3, and stair steps starting in week 4^34)^.

### Week 9 to 16 (phase 3)

Functional dynamic balance training can help further improve neuromuscular control and the kinetic chain between the ankle, knee, hip, and trunk^35)^. In addition, the vestibular and musculoskeletal systems can be challenged by changing surface stability, visual input, task complexity, resistance, and rate of exercise performance during functional dynamic balance training^36)^. Moreover, starting the light and slow speed of plyometric exercises (such as vertical jump, forward double-leg jump, and drop vertical jump) may increase the muscle’s ability for quick contraction, which is a good way to be ready for agility training. In week 12, exercises should include normalization of running (gradually increasing distance and speed to reduce neuromuscular adaptation and recovery time), with outdoor jogging to follow in week 16^37)^.

### Week 16 to 24 (phase 4)

The main goals of this phase are to maximize knee neuromuscular control, muscle endurance, and strength using dynamic functional strength exercises, sport-specific exercises, plyometric exercises, and agility training^38)^. Improved neuroplasticity after ACLR could prevent new injuries during sports participation through exercises specific to sports that include a variety of acceleration, deceleration, turning, cutting movements, running, and jumping^39)^.

## Knee Functions Following ACLR

Knee function is the ability of the knee to perform various types of functional movements. Most patients expect to achieve maximum knee function or at least more than 80% of their initial condition before the injury; therefore, it does not fall into the category of poor knee function after ACLR^40)^. In addition, reduction in knee function is related to the psychological state of fear of re-injury and decreased muscular strength^41)^. Deficiency of the knee extensors, especially after returning to normal daily activities, is associated with smaller knee flexion moments, which might increase the risk of knee reinjury^42)^. Limited ROM, inability to squat, and abnormal gait pattern are particular concerns, given the potentially fatal consequences of knee extensor and flexor weakness.

## Patient-reported Outcome Measure for Knee Function Following ACLR

The perspective of patients about their knees following ACLR is important information to know for clinicians to develop strategies to improve their knee function and quality of life. Patients use patient-reported outcome measures to collect data on their health status and domains that are important to their quality of life, such as symptoms, functionality, and physical, mental, and social health.

Patients can utilize the Knee Injury and Osteoarthritis Outcome Score (KOOS) questionnaire, which self-evaluates knee functions after injuries such as post-ACLR, meniscus tears, cartilage defects, and others^43)^. The KOOS has five subscales: activities of daily living (17 items), knee-related quality of life (4 items), other symptoms (7 items), pain (9 items), and sports and recreation function (5 items). There are different ratings for each subscale, ranging from zero (severe knee difficulties) to 100 (no knee problems)^44)^. On average, the five domains of KOOS had a moderate to high score of construct validity ranging from 0.49 to 0.73 and acceptable interclass correlation scores ranging from 0.78 to 0.91^43)^.

The Lysholm knee score was first described in the orthopedic literature in 1982 and was designed to be administered by physicians to measure outcomes after ACL surgery^45)^. The score places a strong emphasis on evaluating knee instability using self-assessment of knee function and signs of instability. In Lysholm’s score, the maximum score is 100 points, in which 64 or less is unsatisfactory, 65 to 83 is fair, 84 to 90 is good, and 91 to 100 points is excellent^46)^. The Lysholm score had an adequate interclass correlation score of 0.84 and moderate to high construct validity values ranging from 0.55 to 0.68^47)^.

The patient’s subjective evaluation of ligament examination, symptoms, range of motion, and knee function is the basis for the International Knee Documentation Committee’s (IKDC) subjective evaluation form^48)^. The IKDC has sufficient reliability ranging from 0.87 to 0.98 and has a high construct validity with *r* = 0.68^49)^. An ordinal scoring approach assigns a score from 0 (the lowest level of function or the greatest degree of symptoms) to 100 (no restrictions on regular activities or participation in sports, and are symptom-free)^50)^.

The Tegner Activity Scale (TAS) is a patient-reported outcome measure that quantifies a patient’s level of work- and sports-based activity on a one-item questionnaire graded from 0 to 10. The maximum impairment is represented by level 0, and top athletes are represented by level 10. The patients were instructed to select activity levels depending on their current activity level or sports. It showed strong construct validity (*r* = 0.68) and satisfactory test-retest reliability (ICC = 0.82)^47)^.

## Physical Assessments for Knee Functions after ACLR

### Muscle strength test

Muscle strength is the amount of muscle ability maximally released in both static and dynamic positions. Isokinetic dynamometry performed slowly as concentric knee extension torque (30–60 degrees/second) is currently the gold standard for quadriceps muscle strength testing after ACLR^51)^. The peak knee extension torque score was calculated by dividing the affected and unaffected limbs as limb by the limb symmetry index (LSI). Isokinetic testing at a slower speed can identify greater quadriceps strength impairments than at a quicker speed^52)^.

### Hop tests

Hop tests are commonly used to assess RTS that involve muscle strength, neuromuscular control, and dynamic knee stability^53)^. There are variations in the hop test, such as the triple 6-m timed hop, triple crossover hop for distance, hop for distance, and single-legged hop for distance. The most common and widely acknowledged test is the single-leg hop for distance, a successful test with a score of >90% LSI^54)^.

### Drop vertical jump test

The drop vertical jump measures a subject’s leg strength and power in which they must jump as high as possible after dropping a box^55)^. A tool with a box height of 20–100 cm was used for the test. The subjects were asked to step onto the box to start the test, after which their hands were placed on their hips. The subject quickly exited the box, performed a maximal vertical jump, and landed on the mat with both feet. There is sufficient time between several trials to allow for the measurement of ground response forces, flight time, jump power, jump height, and contact time^56)^.

### Star excursion balance test

The star excursion balance test (SEBT) involves a series of lower-extremity reaching exercises in eight different directions as part of a dynamic stability test. After ACLR, the dynamic stability of the lower limb was evaluated using the SEBT, with satisfactory inter-rater reliability ranging from 0.86 to 0.92^57)^. The subject was asked to start the test by standing barefoot in the middle of eight strips of measuring tape that were each marked 1 cm and 45° apart on the ground. The subject was then directed by the examiner to reach the farthest distance of each tape using the free leg while maintaining contralateral leg stability. The score was determined by averaging the results of the three assessments^58)^.

## Risk Factors of Poor Knee Functions After ACLR

### Age

Age is an internal risk factor for impaired knee function after ACLR. Studies show that two years following ACLR, the knee functions measured by the five KOOS subscales improved, yet the improvement in knee-related QOL was less pronounced in older patients^59)^. ACLR in older patients is associated with higher complication rates than that in younger patients and reported at least one postoperative complication (such as venous thromboembolism, deep vein thrombosis, and pulmonary embolism) within 90 days after ACLR^60)^.

### Gender

A previous study reported that male patients made more progress than female patients in terms of knee pain, symptoms, and ADL^59)^. Male patients reported less knee pain than did female patients, suggesting that male patients were less likely to admit pain to show a strong attitude. Male patients showed more endurance when suffering from knee pain across various activities than female patients^61)^. According to previous research, women experience less progress in their ability to perform everyday tasks with their knees than men and this may be related to women’s lower levels of rehabilitation motivation and knee muscle strength^62)^.

### Time from injury to surgery

It was discovered that patients’ knee symptoms, activities of daily living, sports, and quality of life improved less after two years of ACLR if surgery was delayed after the injury^59)^. Moreover, as the interval between injury and ACLR lengthened, the patients’ knee function improved less. This may account for its negative correlation with isolated meniscal tears, subsequent femoral notch stenosis, and chondral injury^63,64)^.

### Graft type

It is important to consider the graft type used to replace a ruptured ACL. A previous study found that the patellar tendon graft for ACLR received less knee pain relief than the hamstring tendon graft after two years of follow-up^59)^. Considering the patella tendon graft’s advantages in terms of graft stability, tunnel size, and RTS quality, it was still possible for donor site problems, including anterior knee pain and patella fracture^65,66)^. An earlier study suggested that hamstring tendon grafting enhanced daily tasks, including the capacity to kneel and kneel walking, which are frequently used in cleaning, construction labor, childcare, and religious activities that call for kneeling^67)^.

## Definition of Post-traumatic OA following ACLR

Post-traumatic osteoarthritis (PTOA) is a type of OA that occurs following joint injury such as intra-articular rupture, ligament injury, or other cartilage (articular or meniscus) impairment inside a joint^68)^. Despite idiopathic OA, PTOA is a source of functional impairment in a disproportionately young population owing to the higher probability of initial injuries in younger people. Furthermore, PTOA frequently has a recognized “starting point,” which suggests that therapies might theoretically be executed early to prevent disease development^11)^.

According to statistics, more than 50% of ACL injuries lead to PTOA^69)^. Grade III or IV radiologic alterations in the Kellgren-Lawrence grading system are approximately five times more prevalent after ACL damage than in contralateral knees without an ACL history^70)^. Within hours following ACL rupture, elevated levels of C-telopeptide fragments in synovial fluid (a biomarker of cartilage degeneration) and matrix metalloproteinases (catabolic enzymes implicated in extracellular matrix breakdown) occur within hours of ACL rupture^71)^.

## Physical Assessment of OA following ACLR

Following ACLR, knees with PTOA showed persistent muscle atrophy and worse knee function^72)^. It should also be noted that joint tissues do not appear to be the only cause of pain and dysfunction in patients, but the periarticular muscles are also implicated in the clinical presentation of many patients^73)^. Patients with knee PTOA frequently present with impaired force-generating capacity in the quadriceps, which can be related to muscle atrophy as well as muscular inhibition, which is a significant predictor of physical function in patients with knee OA^74)^.

## Radiological Assessment of PTOA after ACLR

The morphological manifestation of PTOA is local cartilage injury caused by chondrocyte death and matrix dispersion^75)^. Natural changes reported in a non-injured knee with aging include structural modifications of type II collagen and diminished capacity of chondrocytes to repair the damaged extracellular matrix^76)^. OA changes were more commonly found on radiographs in individuals who underwent ACLR than in their uninvolved contralateral knee following surgery^77)^. Over 70% of patients were classified as asymptomatic PTOA when assessed using KOOS and IKDC-subjective scores, while only 28% prevalence of PTOA was observed after 22 years of ACLR with patella graft^78)^. In addition, patients with less than normal knee extension at discharge from physical therapy and loss of normal knee flexion were also important variables, with 2.02–3.84 times increase in the risk of developing PTOA at the 20-year follow-up^79)^.

## Risk Factors of PTOA following ACLR

### Age

A previous study reported that ACLR at the age of over 30 years predisposes to PTOA^80)^. Pathological changes in the knee joints are common in older people and can worsen after ACL injury and surgery. These changes include the development of osteophytes, thickening of the subchondral bone, degeneration of the knee ligaments and menisci, destruction of articular cartilage, and varying degrees of synovial inflammation^81)^. According to an animal study, older mice showed fewer cartilage metabolism genes and higher inflammatory cytokine activity than younger mice, which is likely to be a factor in PTOA^82)^.

### Overweight

Overweight individuals are subjected to higher mechanical loads at the time of injury, which increases the likelihood of internal derangement^83)^. Obesity is a well-known risk factor for meniscal and chondral lesions, as well as future osteoarthritis^84)^. Patients with a BMI greater than 30 had a considerably higher chance of developing radiographic osteoarthritis after arthroscopic partial and complete meniscectomies^85)^. These patients were also more likely to report higher levels of knee pain and lower knee function scores.

### Meniscus tear

Meniscal tears occur in half of ACL injuries and are a significant element that may contribute to the advancement of PTOA after ACLR^86)^. Compared with individuals with isolated ACL injuries, patients with meniscal tears are more likely to develop radiographic OA following ACLR^87)^. Damage to the meniscus reduces the joint’s ability to absorb force on the cartilage of the femoral bone on the cartilage of the tibia during movements in daily activities and sports^88)^. The meniscus, as a physiologically active tissue, may produce a variety of soluble enzymes and inflammatory mediators in response to trophic damage, which may hasten the degeneration of neighboring cartilage^89)^.

### Cartilage defects

Knee trauma or injury on the ACL increases the force’s impact on cartilage tissue that starts chondrocyte necrosis and apoptosis; thus, over half of the patients also have articular cartilage degradation of the medial and lateral femoral condyles^90)^. The mechanical impact causes increments of chondrocyte expression of matrix-degrading enzymes and inflammatory cytokines, resulting in chondrocyte death^91)^. The low remodeling potential and subchondral destruction after ACL injury occur because of the disruption of the bone resorption and creation balance, which may develop PTOA on the articular chondral surface following ACLR^92)^.

## Preventive Strategies of Poor Knee Functions and Osteoarthritis after ACLR

### Pre-operative program

Prior investigation showed that pre-operative exercises could enhance self-reported knee function, positive outcomes in RTS, and prevent PTOA^93)^. Furthermore, pre-operative exercises could minimize lower limb neuromuscular function degradation after surgery^94)^. There are two phases of pre-operative treatment of ACLR^95)^. The initial phase starts from day 1 to 4–6 weeks after injury to restore full knee ROM, resolve inflammatory symptoms, and co-contraction and muscular control. The next phase, on average, starts from 4–6 weeks to 12 weeks after injury and focuses on a progressive exercise program for muscular strength and neuromuscular function recovery. Additionally, plyometric exercises (e.g., single leg hops with soft landings) and advanced neuromuscular (e.g., perturbation, balance, stability, proprioceptive exercises) must be considered. Greater-dose pre-operative exercises should not begin before the initial resolution of disability (approximately 2-3 months), depending on functional and tissue recovery and any concurrent injuries^96)^.

### Comprehensive rehabilitation

Comprehensive rehabilitation after ACLR is crucial for a successful operative outcome. A faster rehabilitation start after the ACLR has not been associated with negative outcomes^97)^. Therefore, it is safe for patients to start weight-bearing immediately after surgery, flex their knees to 90 degrees, and do closed-chain-strengthening exercises^2)^. Three weeks after surgery, it was safe to add eccentric quadriceps muscle strengthening and isokinetic hamstring muscle strengthening^98)^. Strength and range of motion exercises must be done in addition to neuromuscular workouts. Single-leg cycling may be advantageous for maintaining heart fitness^99)^.

### Body weight control

Obesity is also linked to decreased functional mobility and is a known risk factor for OA^84)^. New weight gain will exacerbate the risk of PTOA from an earlier joint injury. Interventions focused on maintaining a healthy weight for those with an intra-articular knee injury appear reasonable. Evidence demonstrating the difficulties of continuing and the high expense of multi-component weight-loss regimens supports maintaining a healthy weight rather than relying on additional weight-loss therapies^100)^. Still, secondary prevention programs’ effectiveness in preserving a healthy weight requires further data on physical activity engagement, dietary habits, and individual perspectives^101)^.

### Return to sport (RTS) consideration

*a. Physical capacity*

Before starting the RTS phase (>20 weeks), the patient must have fulfilled the conditions as follows: no pain or swelling; full knee ROM; limb symmetry of 75% to 85% with isokinetic testing, handheld dynamometry, or 10-repetition maximum examination of the gluteus maximus, gluteus medius, quadriceps, and hamstrings; Y-balance test with 90% limb symmetry; and ten repetitions of a single-leg squat through 60° ROM and/or a single-leg stork balance test^102)^. This rehabilitation stage aims to maintain advanced strengthening, return to running, begin a plyometric program, initiate an agility program, and incorporate certain sport-specific motions. Patients will begin with submaximal efforts in all these programs, beginning with straight plane and 2-leg movements^103)^. The patient should be evaluated for both qualitative and quantitative outcomes. The physical therapy goal regarding landing mechanics is to reduce hip drop, hip internal rotation, knee valgus, heavy or stiff landings, and loss of balance. Re-evaluating of measurable indicators is required, preferably every 4 to 6 weeks as the patient advances through the initial stages of RTS, leading to the patient developing multiplanar movements and maximal-effort plyometrics after 80% to 90% limb symmetry with hop testing and limb symmetry with strength testing^104)^. By this stage, the patient should begin the return-to-play progression^102)^:

1. On-field plyometric drills and agility, followed by non-contact practice
2. Contact or a full practice
3. Full gameplay

Athletes who failed to meet adequate physical ability before returning to professional sport had a fourfold increased risk of ACL graft rupture and potentially impaired knee function^105)^.

*b. Proper time of RTS*

The incidence of ACL reinjury was lowered by 51% each month. RTS was postponed until 9 months following surgery^106)^. Considerations of biological variables of tissue healing have a role in deciding the timing for RTS using a conservative rehabilitation strategy. Many experts advocate returning to full exercise after 9 to 12 months since graft tissue is subject to necrosis, revascularization, and remodeling^107,108)^. Allografts normally undergo revascularization and ligamentation over a 12-month period^109)^. After 6 to 12 months, vascularity and fiber pattern could return to normal. The main objective is to restore baseline joint homeostasis, neuromuscular control, strength, and proprioception in the knee, which can take up to two years in some individuals^110)^. The latest research indicates that the RTS decision following ACLR should ideally be postponed from the 6-month timeframe to at least 12 months post-surgery^97)^.

*c. Psychological readiness*

Patient psychology has emerged as a significant concern in RTS following ACLR. Kinesiophobia is the presence of movement alterations contributed by a psychological factor of movement-related fear linked to worse outcomes of knee functions^111)^. Patients failed RTS due to a concern of a new injury, lack of trust in the knee, and inadequate knee function. Injury time lost and returning to pre-injury activity were closely associated with psychological readiness for RTS. A recent consensus statement on sports-related injuries proposed an integrated approach incorporating physical, psychological, social, and environmental factors to optimize rehabilitation programs^112)^. Two social domain themes and three contextual domain themes can increase a patient’s psychological readiness for a RTS^113)^. The first social domain is the social support from family, friends, teammates, coaches, physical therapists, and other medical staff. The transfer of resources such as informational or emotional support recovery expectations, negative emotions, and risk assessment on RTS often comes with greater adherence, resilience, confidence, and self-motivation. Second, the goal setting on physical care. A robust therapeutic partnership in which athletes’ specific aims and beliefs were valued resulted in pleasant rehabilitation experiences and increased confidence in healthcare practitioners^114)^.

The first contextual domain theme is environmental influences, such as insufficient time or equipment, private activity preferences, and making rehabilitation comforting or demanding, which can support behaviors associated with greater obedience to rehabilitation, self-motivation, and autonomy. The second domain is sports culture. Athletes are considered premature RTS due to high social pressure from colleagues and a risk-taking culture throughout sports. The last domain is individualization. A need for social support, individual coping techniques, the choice to maintain or avoid social duties, and contextual factors all impact treatment adherence. Recovery goals varied by gender and age, with males and younger athletes expressing higher RTS expectations and pressures than women or adults^113)^. The ACL-RSI (ACL-Return to Sport after Injury) has assessed psychological preparedness to RTS. Yet, there needs to be more evidence of its application in young athletes. Following, scores at a 6-month postoperative exam improved. According to a recent study, an altered score of 13.4 points represents a minimum significant change in psychological awareness at the group level^115)^.

## Conclusion

Surgery procedures of ACLR are enormously complex, and there are still challenges in determining appropriate strategies for achieving each goal. Generally, the ACLR procedure needs to choose the graft types to replace the ruptured ACL, which may have its advantages and disadvantages for future knee functions. The BPTB has still become the gold standard graft. Nonetheless, the hamstring tendon is the most commonly used by orthopedic surgeons, where it has less anterior knee pain and results in low donor site morbidity because of the minor incision procedure. There are two general assessments to determine poor knee function that include patient-reported outcome measures (i.e., KOOS, Lysholm’s score, IKDC-subjective questionnaire, and TAS) and physical assessments (i.e., muscle strength test, hop tests, drop vertical jump test, and star excursion balance test). At the same time, there is an additional radiological assessment besides the physical assessment to diagnose the PTOA.

The successful ACLR program has been challenging for decades to achieve the preinjury level of knee function and avoid PTOA. Early surgery after injury and in younger patients are essential factors to consider preventing adverse outcomes in knee symptoms, daily living functions, and quality of life following ACLR, while other factors such as patients’ gender and graft types are also risk factors for adverse knee pain. In addition, more than half of ACL injuries lead to PTOA, and some factors during ACLR should also be considered risk factors for PTOA. Older and overweight patients are risk factors for PTOA because of cartilage degeneration and high mechanical force damaging the sub-chondral of femoral and tibial bones, respectively. Patients with ACL injuries that cause meniscus tear and cartilage defects had a higher risk of getting PTOA than those with a sole ACL injury after the ACLR. Several strategies can prevent poor knee function and PTOA after ACLR, including a pre-operative program, comprehensive rehabilitation, body weight control, and return to sport consideration based on physical capacity, proper time, and psychological readiness.

## Conflicts of Interest

No commercial party with a direct or indirect interest in this study will offer valuable things to the authors or affiliated organizations.
